# Twelve Tips for using Learning Curves in Health Professions Education Research

**DOI:** 10.12688/mep.19723.1

**Published:** 2023-11-07

**Authors:** Neva Howard, Roger Edwards, Kathy Boutis, Seth Alexander, Martin Pusic

**Affiliations:** 1 Pediatrics, University of Colorado School of Medicine, Aurora, Colorado, 80045, USA; 2 Health Professions, MGH Institute of Health Professions, Boston, MA, 02129, USA; 3 Pediatrics, University of Toronto, Toronto, Ontario, M5G 1X8, Canada; 4 School of Medicine, The University of North Carolina at Chapel Hill, Chapel Hill, North Carolina, 27599, USA; 5 Pediatrics, Harvard University, Boston, Massachusetts, 02115, USA

**Keywords:** Learning Curves, Medical Education, Data Modeling, Curricular Design, Curricular Evaluation, Skill Development

## Abstract

Learning curves can be used to design, implement, and evaluate educational interventions. Attention to key aspects of the method can improve the fidelity of this representation of learning as well as its suitability for education and research purposes. This paper addresses when to use a learning curve, which graphical properties to consider, how to use learning curves quantitatively, and how to use observed thresholds to communicate meaning. We also address the associated ethics and policy considerations. We conclude with a best practices checklist for both educators and researchers seeking to use learning curves in their work.

## Introduction

Although developing skills over time is a core part of health professional education, measurement of new skills and descriptions of how to attain these skills are often ill-defined ( Howard *et al.*, 2021). Learning curves are a robust way of describing and measuring skill development ( Howard *et al.*, 2021; Pusic *et al.*, 2015). The three elements of a learning curve are a vertical axis representing a measure of the desired knowledge or ability (achievement), a horizontal axis that represents the amount of time spent in effortful learning, and a linking mathematical function that describes how effort and achievement are related ( Pusic *et al.*, 2015). Some phases of learning are more difficult than others, but with sufficient perseverance, the learner follows a predictable path to achieve the desired level of expertise. The shape of the curve will depend on the intrinsic difficulty of the skill to be acquired, the individual learner, as well as the learning context. Close examination of the learning curve can visually and mathematically describe these complex, interrelated factors ( Ramsay *et al.*, 2001).

A considerable amount of linguistic confusion exists when discussing learning curves. First consider a “steep learning curve”, which is colloquially meant to refer to a skill which is difficult to master in the way a steep hill would be difficult to climb. However, mathematically and graphically speaking, the steeper the learning curve, the better. This indicates that the slope, representing the amount of learning per unit of effortful time invested, is maximized, with learners showing rapid learning gains. In fact, making learning efficiency (or lack thereof) explicit is a clear benefit of the learning curve representation. Also potentially confusing, the term learning curve or learning growth over time is used to represent multiple complex learning contexts that are prevalent in Health Professions Education (HPEd): educational frameworks ( *e.g.* competency based medical education, milestones), instructional designs ( *e.g.* deliberate practice), expertise development ( *e.g.* Dreyfus & Dreyfus model) and an application of a mathematical theory ( *e.g.* growth curve modeling) ( Boscardin *et al*., 2022; Leppink, 2015; Pusic *et al.*, 2016).

**Figure 1.**
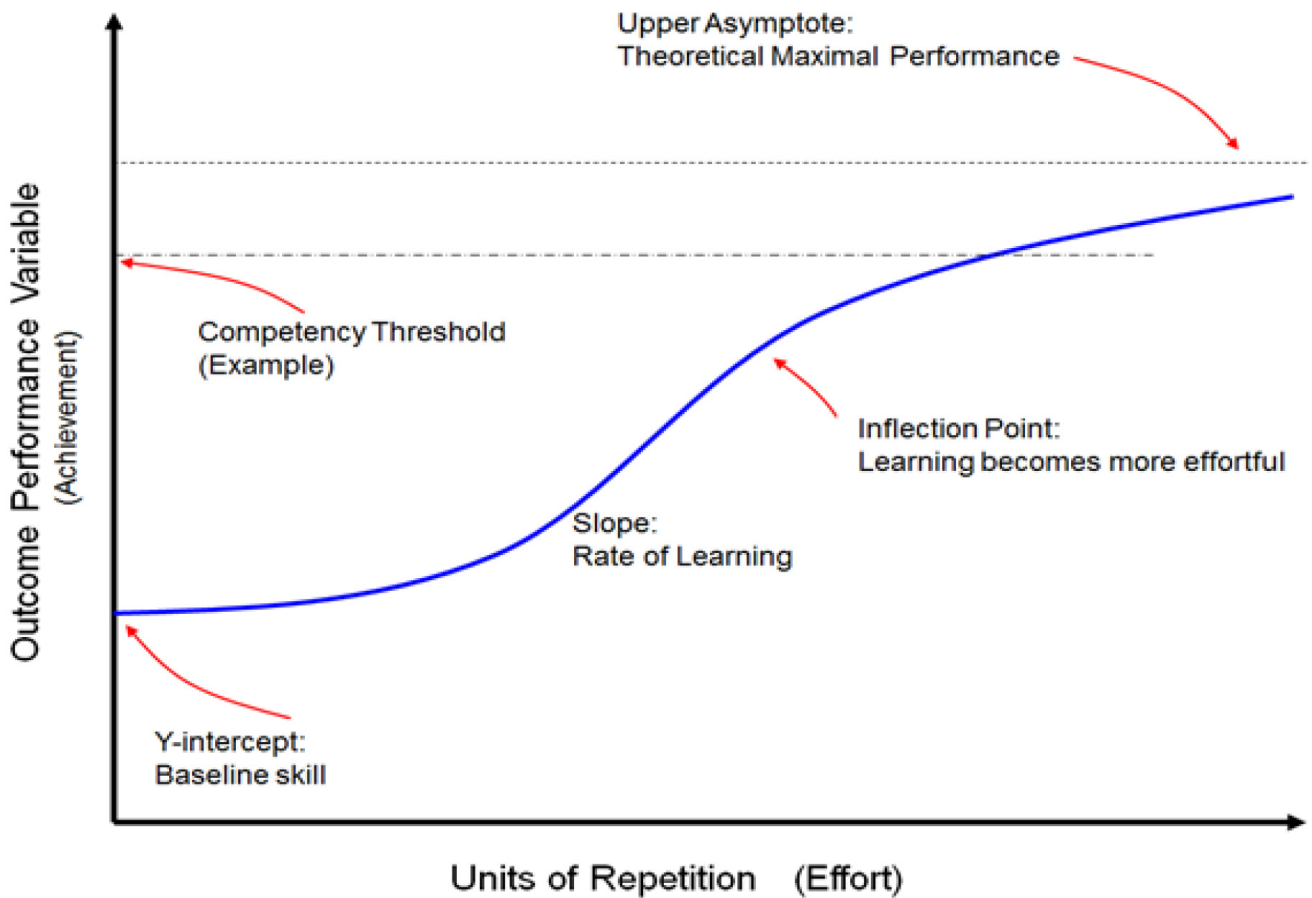
The learning curve demonstrates the relationship of effort (X-axis) and achievement (Y-axis). The Y-intercept represents a learner’s baseline skill and is followed by a relatively flat latent phase of growth at the beginning of the curve as the learner orients to the learning environment. Learning then accelerates, producing a slope that represents effortful learning. At the next inflection point, basic skills have been learned and further acquisition of expertise becomes more effortful. The competency threshold is a predefined level of achievement. The curve represents how many repetitions are necessary for an individual (or group) to demonstrate competency. Considerable effort can push the learner beyond competency to greater levels of expertise, with the upper asymptote being the theoretical maximum for which the true expert strives. Note that the blue line can represent an individual learner or a group average. Refer to Table 1 for more detailed definitions. This figure is an original figure produced by the authors for this review article.

From a learner’s perspective, the typical learning curve represents core educational truths. First, the learner has some baseline knowledge or skill, represented by the *y-intercept*. Next, learning does not always start with a phase of growth. Instead, there is often a *latent phase* where the learner orients to the learning environment without improving in terms of their Y-axis (learning) metric; however, once all necessary elements are in place, learning takes off, *accelerating* at perhaps its greatest pace in terms of how much gain in performance occurs per unit of effort. Once the simpler elements of a domain are learned, however, the curve demonstrates an *inflection point* where gains in performance become proportionately more difficult to achieve (colloquially referred to as the law of diminishing returns). Attaining a mastery learning standard after this inflection point is hard won, requiring significant effort ( Ericsson, 2015). Finally, thresholds of proficiency, competency, or mastery can be developed and represented as horizontal lines intersecting the learning curve (See Figure 1). Please see Table 1 for definitions of all learning curve elements.

**Table 1.** Definitions and recommended best uses of learning curve elements. Please use as a reference for definitions throughout the paper. Adapted from Howard *et al.*, 2021 with permission ( Howard *et al.*, 2021).

Learning curve feature	Specific element of learning curve	explanation
*General* Tip one	Learning curve	Graphical, tabular, or statistical analysis comprised of at least three data points which represent an individual student’s achievement versus learning effort over time.
*Y-axis* Tip two	Y-axis of learning curve	The achievement or performance of an individual or group. The Y-axis should be the most valid representation possible of quantitative performance within your educational project.
	Full vertical scale	The full vertical scale from no skill to maximum possible performance. Without the full scale, the display can result in misleading interpretations by visually exaggerating findings ( Tufte & Graves-Morriss, 1983).
*X-axis* Tip three	Repeatable unit on x-axis	A repeatable and measurable unit of student effort during the learning process. The unit may consist of a repetition such as the number of cases, sessions, or trials, or consist of a continuous measure of time such as days, weeks, or months.
	Repetition spacing specified	X-axis unit spacing in time is clearly specified. This does not have to be regular but must be reported in a reproducible manner. For example, a daily session with a variable number of repetitions is a full report of spacing. Reporting five sessions in six weeks chosen by the participant is suboptimal (sessions could be five days in a row vs. once per week).
	Forgetting curve	The data points on a curve which represent student performance after active learning interventions are complete. Ideally this includes a data point further out in time than the regular testing interval. Together a learning curve and a forgetting curve may be referred to as an experience curve.
	Post-test or retention test	Tests given after the learning phase that are not part of the learning curve. No interventions occur between the learning curve and a post- or retention test. A post-test is typically given shortly after the learning phase while a retention test is given after a longer delay.
*Deliberate practice*	Feedback exposure	Effortful practice of a task/skill combined with expert level feedback over many repetitions and/or exposures.
	Repetitions increase in difficulty	Difficulty increases as a learner demonstrates mastery of easier tasks. If the tasks are computerized this would be a form of computer adaptive testing or learning, allowing learners to continue along the path of effortful practice after some tasks are mastered.
*Graphing* Tips six, seven	Group learning curve	A learning curve representing the average performance across members of the group. A group learning curve should include a measure of statistical variation along the curve.
	Overlaid individual learning curves	A single graph showing the learning curves of each member of a group. This plot informs the individual variation within a group and shows the individual paths taken.
	Stacked learning curves	A graph with several learning curves, either individual or group curves, with a common X-axis which shows how variables independently change for the same case/example. The graph can consist of several curves on a single graph or multiple graphs stacked.
*Linking function* Tips four, five	Linking function	A mathematical function that describes how the X-axis variable (effort) is related to the Y-axis variable (performance).
	Linking function – group level function	The mathematical function that links the group X-axis variable (effort) with the group Y-axis variable (performance). This includes any statistical analysis of the incremental improvement from the beginning to the end of the curve or learning experience. This can be done using a repeated measures ANOVA, or with a function “fit to the data” with an equation such as power law, exponential, logarithmic, or linear.
.	Linking function - hierarchical linear modeling (HLM), growth curve modeling	A linking function using group level data to estimate the performance of an individual within the group. The HLM or growth curve modeling can predict how much deliberate practice is necessary for competency of an individual, or what level of intervention is needed, based on an individual’s coordinates in the system ( Leppink, 2015; Mema *et al.*, 2020; Pusic *et al.*, 2016).
*Competency frameworks* Tips eight, nine	Boundary line (mastery or remediation line)	Competency or remediation boundary line drawn on the learning curve, or a clearly specified boundary criterion that would allow someone to draw the boundary line. In addition to measuring mastery and competency this line can signal a need for intervention to reorient the student to the predicted learning curve.

What follows are twelve tips for using learning curves well in HPEd and research.

## In general: the x- and y-axis

### Tip one: decide whether a learning curve can help in your learning/teaching mission

Learning curves have a variety of applications in HPEd. Learning curves represent learning, or achievement over time. Single assessments in time, such as an isolated Observed Structured Clinical Exam (OSCE) or a multiple-choice exam, do not capture learning that has occurred along the way but instead function as more summative, cross-sectional, end-goal assessments. Having a final exam may seem sufficient to judge competency; however, much information is lost about the path taken when evaluating a learner only at the end of their training. This may be particularly relevant when considering remediation or evaluating competency to accelerate a learner. Thus, whenever the *learning path* is of interest, the learning curve can be a powerful representation of student achievement, the effectiveness of learning interventions, and where more scaffolding might be useful.

Formative quantitative assessments for learning, monitored over time with accompanying feedback, are ideal to represent with a learning curve. For example, situations of deliberate practice in authentic settings are some of the most robust applications of learning curves ( Anders Ericsson, 2008; Pusic *et al.*, 2012). With deliberate practice, there are consistent opportunities for repetitive practice of a particular skill, such as reading an X-ray, ( Pusic *et al.*, 2011) tying surgical knots, ( Hsu *et al.*, 2015; Thiyagarajan & Ravindrakumar, 2016) resuscitation simulation, ( Abelairas-Gómez *et al.*, 2016) and even measures of intrinsic motivation or mindset ( Li & Bates, 2020; Murayama *et al.*, 2013; Paunesku *et al.*, 2015; Richards *et al.*, 2013). Designating a quantitative representation of learner achievement, however, is critical for being able to graph the learning curve. The quantitative measure of achievement should be one that is accurate and sensitive to improvement over time when evaluated in frequent, formative assessments. Examples include time of completion for a surgery ( Favre *et al.*, 2016), a dichotomous outcome such as reading an ECG as normal or abnormal ( Hatala *et al.*, 2019), or an Objective Structured Assessment of Technical Skills (OSATS) score for a simulation ( Hernandez *et al.*, 2004), each being measurable outcomes that should change as learners develop. If these variables exist and can be measured in a given learning context, a learning curve may be appropriate. For examples of how one can apply learning curves to educational work see Table 2. An important limitation of learning curves is that they work best where a quantifiable reference standard can be specified. More research and/or ingenuity is required to apply it to a “fuzzier” task such as taking a psychiatric history.

**Table 2.** Examples of learning environments and corresponding time-based learning effort and outcome performance variables needed to graph a learning curve.

Learning context	Time-based measurements: learning effort (X-axis)	Performance or achievement (Y-axis)
Surgical knot tying task practice ( Thiyagarajan & Ravindrakumar, 2016)	Knot tying sessions	Number of knots tied, time to tie, frustration score, difficulty score
Surgical Simulation	Training Episodes	Time, economy of movement, wall collisions
Complete surgeries ( Hernandez *et al.*, 2004)	Cases	Objective Structured Assessment of Technical Skills (OSATS)
Core competency acquisition in medical school ( Thompson & Rogers, 2008)	Bi-yearly assessments	Self-assessment scores
New technology use, *e.g.* GlideScope use in the Emergency Department ( Ahn *et al.*, 2016)	Sessions of GlideScope use for laryngoscopy	Critical components checklist score
X-ray interpretation ( Pusic *et al.*, 2011)	Individual x-rays read	Accuracy of x-ray reads as normal or abnormal compared to a gold standard
Cognitive clinical skill development: treatment of hyperacute stroke thrombolysis with alteplase ( Chong, 2016)	Code stroke cases seen	Door to needle time, CT (Computed Tomography Scan) to needle time, post-thrombolysis bleeds

### Tip 2: choose how to represent performance/achievement on the Y-axis

**Figure 2.**
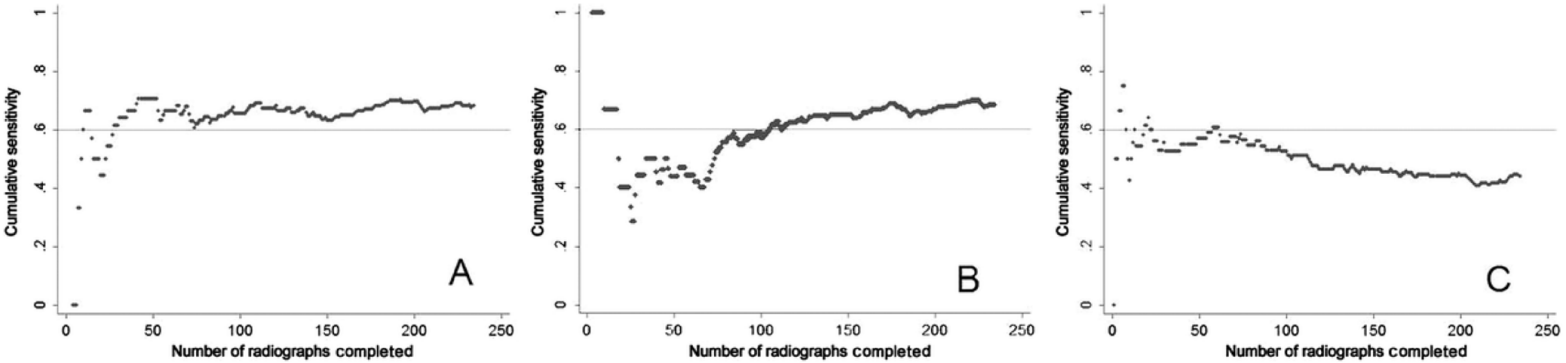
Reproduced with permission from Pusic *et al.*, 2011 ( Pusic *et al.*, 2011). In the cumulative learning curves above, the expert (Learner **A**) achieved the predetermined competency of 0.6 within 50 reads. Learner **B**, a relative novice, has a distinct learning curve as they learn and improve with every X-ray interpreted. Learner **C** shows a negative slope on the learning curve, indicating some other confounding influence, such as boredom, fatigue, or anxiety.

A measurement of performance or achievement is represented on the Y-axis. For examples, please see Table 2. The measure of performance needs to be an accurate representation of the learner’s level of expertise and should be consistent with the literature on how to define expertise within a given domain. For example, one might use the time taken to read an x-ray as a surrogate representation of increased expertise assuming decreased time represents more expertise; however, it has been found that experts often take more time to read an x-ray than a novice, and this measurement would therefore be flawed ( Pecaric *et al.*, 2017). To determine the validity of an assessment or outcome measure, a group of stakeholders should answer the question “does the chosen assessment actually measure what the researcher/instructor thinks it is measuring?”. For example, duration of surgery is often an outcome used on a surgical simulator based on, in part, underlying justifications that decreased time can represent a mastered surgical skill, decreased risk for anesthetized patients, and higher productivity ( Bjorgul *et al.*, 2011). However, when a complication is encountered or a novel technology or procedure is introduced, surgical duration is less predictive of the learner’s expertise than, for example, their response time to a surgical alarm alerting them to a complication. One way to evaluate the utility of a variable is to compare the performance of novices against experts – if the outcome variable is valid there should be a distinct difference between levels and the learning curve should show that experts do not demonstrate further learning gains as they complete repetitions along the X-axis (see Figure 2).

### Tip 3: choose how to represent effort on the X-axis

**Figure 3.**
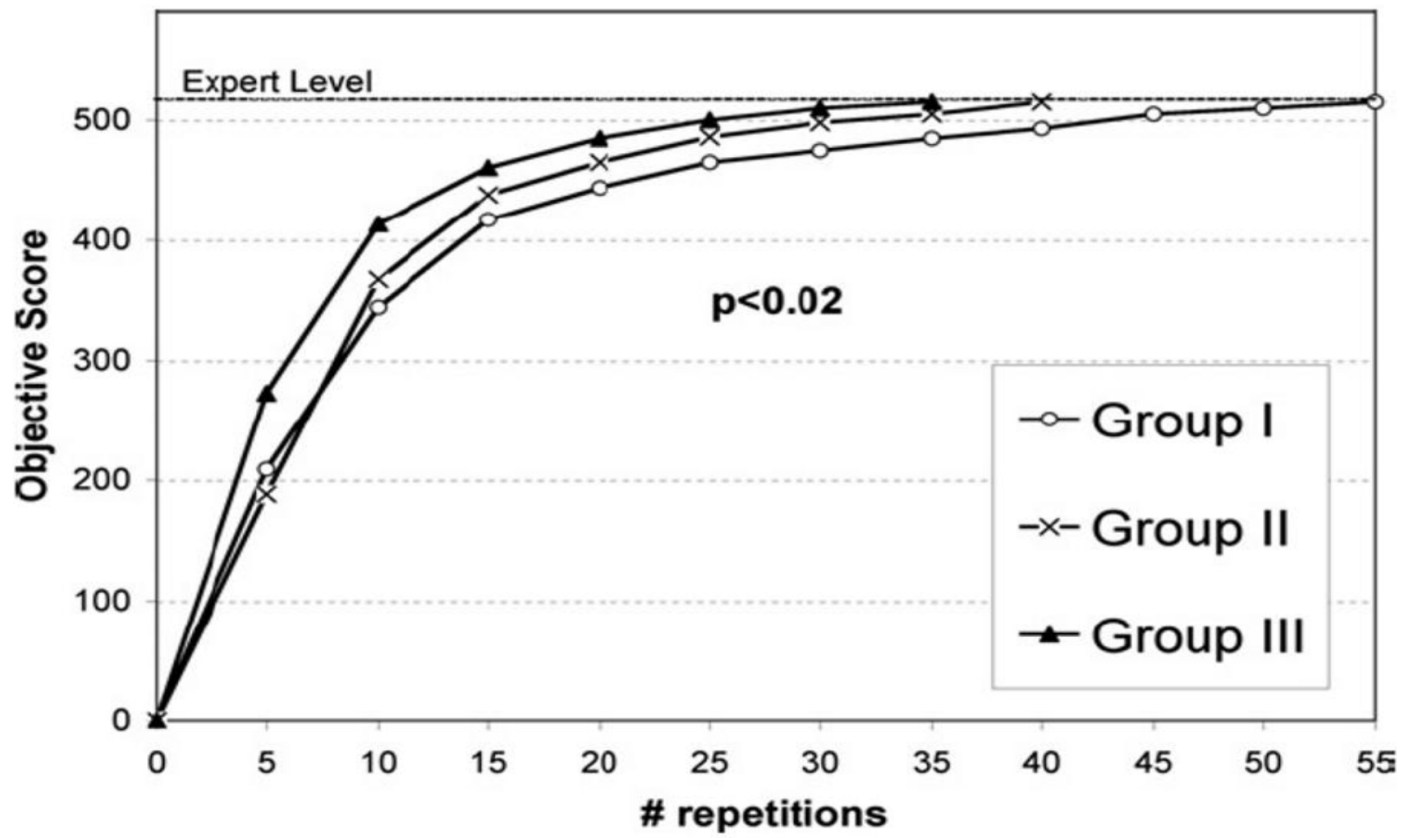
Reproduced with permission from Stefanidis *et al.*, 2007 ( Stefanidis *et al.*, 2007). Each group achieves expert level (or competence), the stopping condition, at separate times. There are different slopes and shapes of the learning curves, denoting unique pathways to the same goal. However, all have achieved a designated standard, ensuring readiness for independent practice.

In creating a learning curve, one must decide how learning effort will be measured in discrete episodes over time. The level of granularity for the X-axis depends on what needs to be represented. Consider these paired examples: a) number of repeated surgeries which describes discrete learning episodes versus b) years in practice which describes more general experience; or a) repetition of specific cases representing deliberate practice versus b) continually unique situations (such as the repeated exposure to presentations seen in apprenticeship training). Higher granularity in the (a) examples of each pair allows for a time-variable representation of how individual trainees or physicians achieve a competency standard when they have individually repeated enough repetitions along the X-axis (See Figure 3). More general measures of effort represented by the (b) examples may be more a representation of one’s learning environment than individual learning.

## Linking function tips

### Tip 4: connect effort with performance quantitatively

Ideally a statistical model, or linking function, should be used to quantitatively describe learning over time. Although statistical modeling could compare the outcome variable (Y-axis) at the beginning and the end, this foregoes any analysis of the learning process in between these points. This is analogous to the loss of information when dichotomizing a continuous variable. The use of statistical models which use all data points on a learning curve, such as growth curve modelling, gives more information about the rate of learning ( Pusic *et al.*, 2016). Multilevel modeling functions compare rates of learning at the group and individual level, allowing intra- and inter- individual and/or group comparisons. As such, multilevel modeling has predictive capacity for individuals and groups within a well-defined system ( Leppink, 2015; Mema *et al.*, 2020; Pusic *et al.*, 2016; Reinstein *et al.*, 2021). Multilevel modeling can evaluate latent variables or covariates that are inferred rather than directly observed, such as motivation or fatigue. Although hierarchical modeling, including multilevel modeling and growth mixed modeling was developed over thirty years ago, their use has been largely limited to education in engineering, biologic and social sciences (i.e. outside of HPEd) ( Ramsay *et al.*, 2001). Considerable statistical expertise is necessary to employ these models, and their use in the health professions education realm is on the rise ( Howard *et al.*, 2021; Leppink, 2015; Thau *et al.*, 2021). Identifying an appropriate statistician early in one’s project can ensure that the data are collected in the appropriate form.

### Tip 5: in research, consider making the learning curve itself the main outcome variable

Often the main outcome variable of an education study or intervention is a cross-sectional post-test, a single snapshot of achievement at one point in time. We argue that *learning rate* should be the main outcome wherever possible. Although learning curves are increasingly common in HPEd, only around half of studies that use learning curves (already a minority of studies) designate the main outcome variable as a learning curve ( Howard *et al.*, 2021). If the y-axis is chosen as the achievement of interest (Tip #2), the rate (slope) of the learning curve, quantified in the linking function, is what most educators are interested in. We are not just interested in how much learning occurred but increasingly we are concerned with how efficiently that learning occurred. How much effort did it take? In a simple pre-post design, the slope between the two data points is a measure of the rate of learning, but one that is much less informative than a learning curve that describes the entire path to competence. As we assume more responsibility for how to achieve outcomes for learners from medical school through fellowship, learning curves offer us insight into remediation, competency, and next steps for any learner.

## Graphing tips

### Tip 6: where possible, graph individual data to communicate overall learning curve impressions

**Figure 4.**
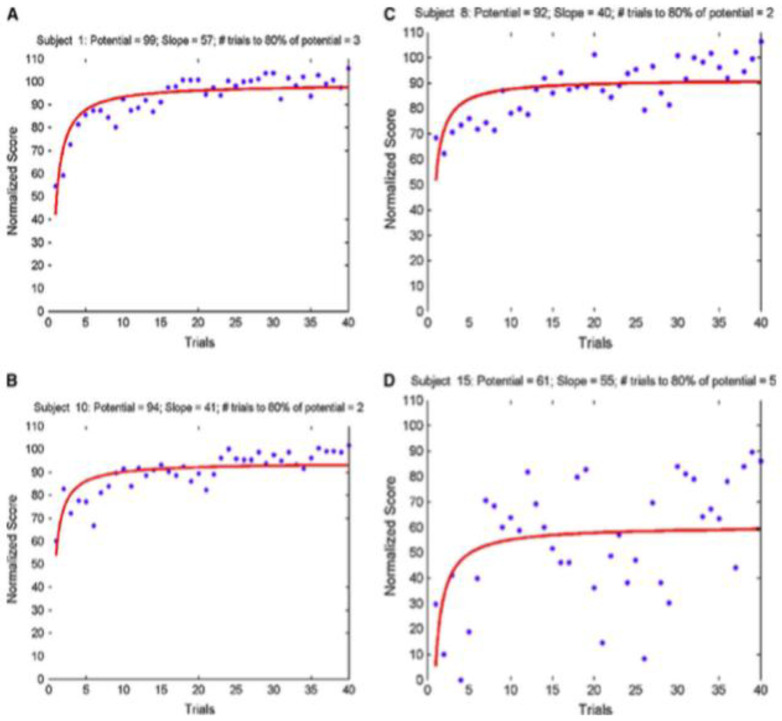
Reproduced with permission from Feldman, Cao, Andalib, Fraser, & Fried, 2009 ( Feldman *et al.*, 2009). Note the immediate visual differences in this demonstration of the use of small multiples. For example, Subject **A** started at a lower score than **B** or **C**, but reached a similar plateau value. Subject **D** showed incredible variability, questioning the validity of the learning curve in comparison to the others.

Raw, individual data, when graphed appropriately, can convey information leading to greater insight into one’s learning intervention. For example, in Figure 4, if only the average individual learning curves were graphed, significant differences between these individuals would be lost. Even though the average lines look similar, one can immediately see a problem with individual D in Figure 4 by putting the individual data side-by-side. This is an example of the visual design principle of small multiples ( Tufte & Graves-Morriss, 1983).

**Figure 5.**
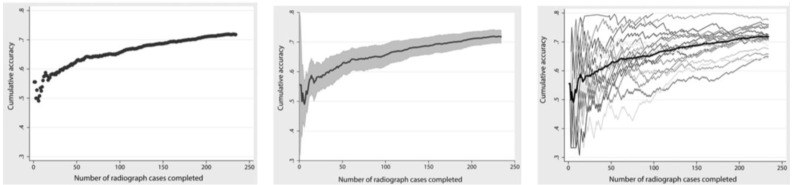
Reproduced with permission from Pusic *et al.*, ( Pusic *et al.*, 2015). Learning curves for 18 residents interpreting ankle x-rays over time with accuracy plotted on the Y-axis as a representation of achievement. The first graph is the average learning curve of the residents, and the second includes the 95% confidence interval. Neither graph conveys the information of plotting all individual curves overlaid in the third graph. There are significant interpretations only glimpsed from the third graph regarding concepts such as competency and remediation. The simple averaged graph could lead the interpreter to very different conclusions.

In the next example, shown in Figure 5, showing an average curve of individuals even with confidence intervals belies the visual information communicated when individual paths are plotted. Only the individual paths communicate information about the meaning of the average curve.

### Tip 7: consider using the “stacking” graphing technique to convey new meaning from your learning curves

Another useful communication technique is the use of stacking learning curves. Comparisons are facilitated when multiple variables are presented either on the same graph or one on top of the other ( Few, 2009). Shapes of learning curves can give both qualitative and quantitative information. In Table 3, different graphs that are stacked on each other can indicate relative expertise level, need for remediation, or a problem with the learning simulator in a quick, easily digestible manner.

**Table 3.** Examples of stacked learning curves (same graph or one on top of the other): each have different outcome variables aligned over a similar X-axis which allows for point-by-point comparisons of the variables and overall shape of the learning curves. This allows for comparison of values for different outcome variables using quick visual analysis.

Graphical representation	Example graphs
The differences in the shapes of the curves demonstrate differences in expertise level.	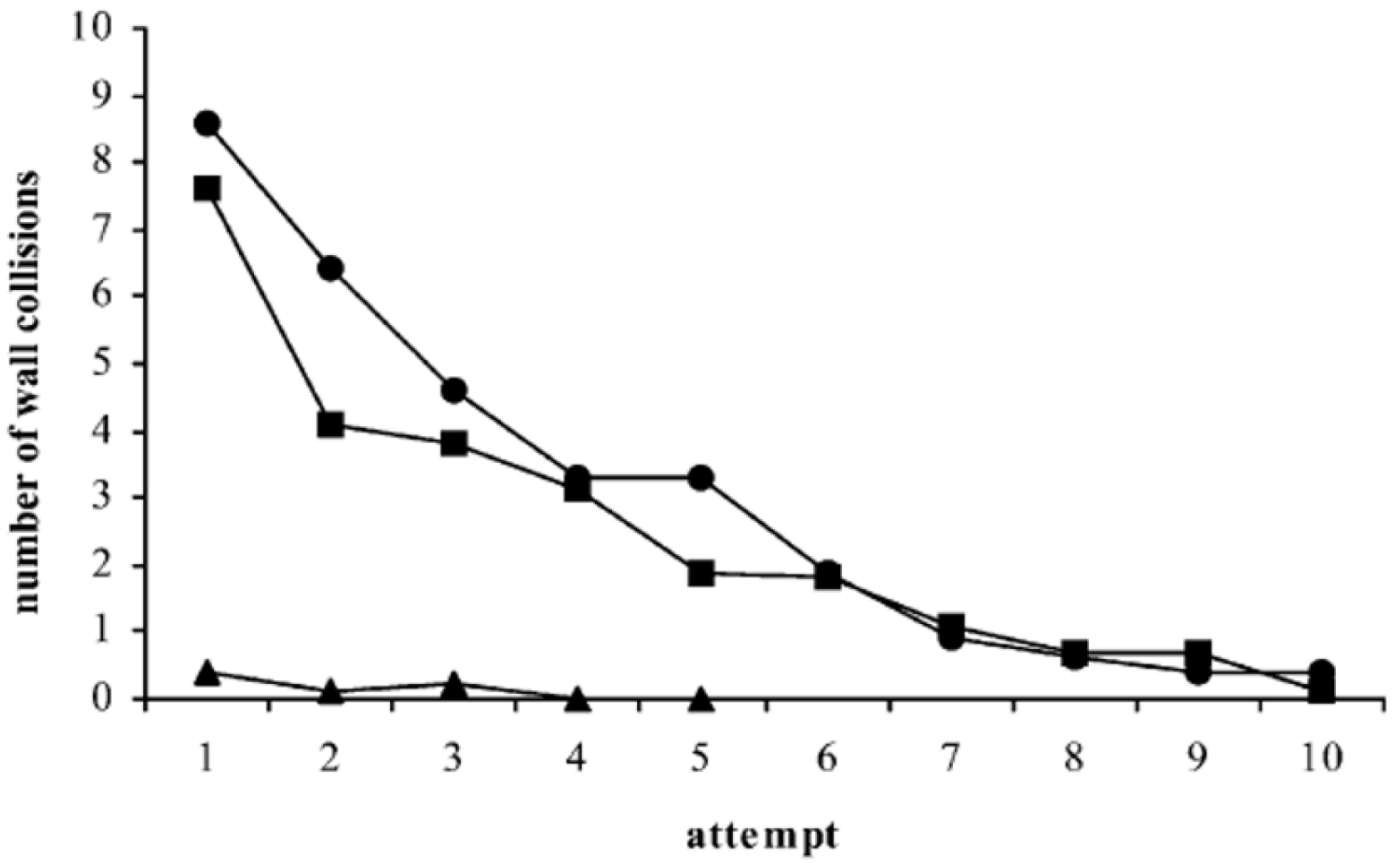 **Figure 6**: *Reproduced with permission from Eversbusch & Grantcharov, 2004* ( Eversbusch & Grantcharov, 2004). Note the flat curve of the experts denoted by triangles compared to residents (squares) and medical students (circles) stacked in the same graph. This supports the validity of the intervention as experts by definition should not demonstrate a learning curve in comparison to novices (medical students circles, residents squares, attending experts triangles).
A flat curve below the proficiency threshold (increased time in this case) can indicate a need for remediation.	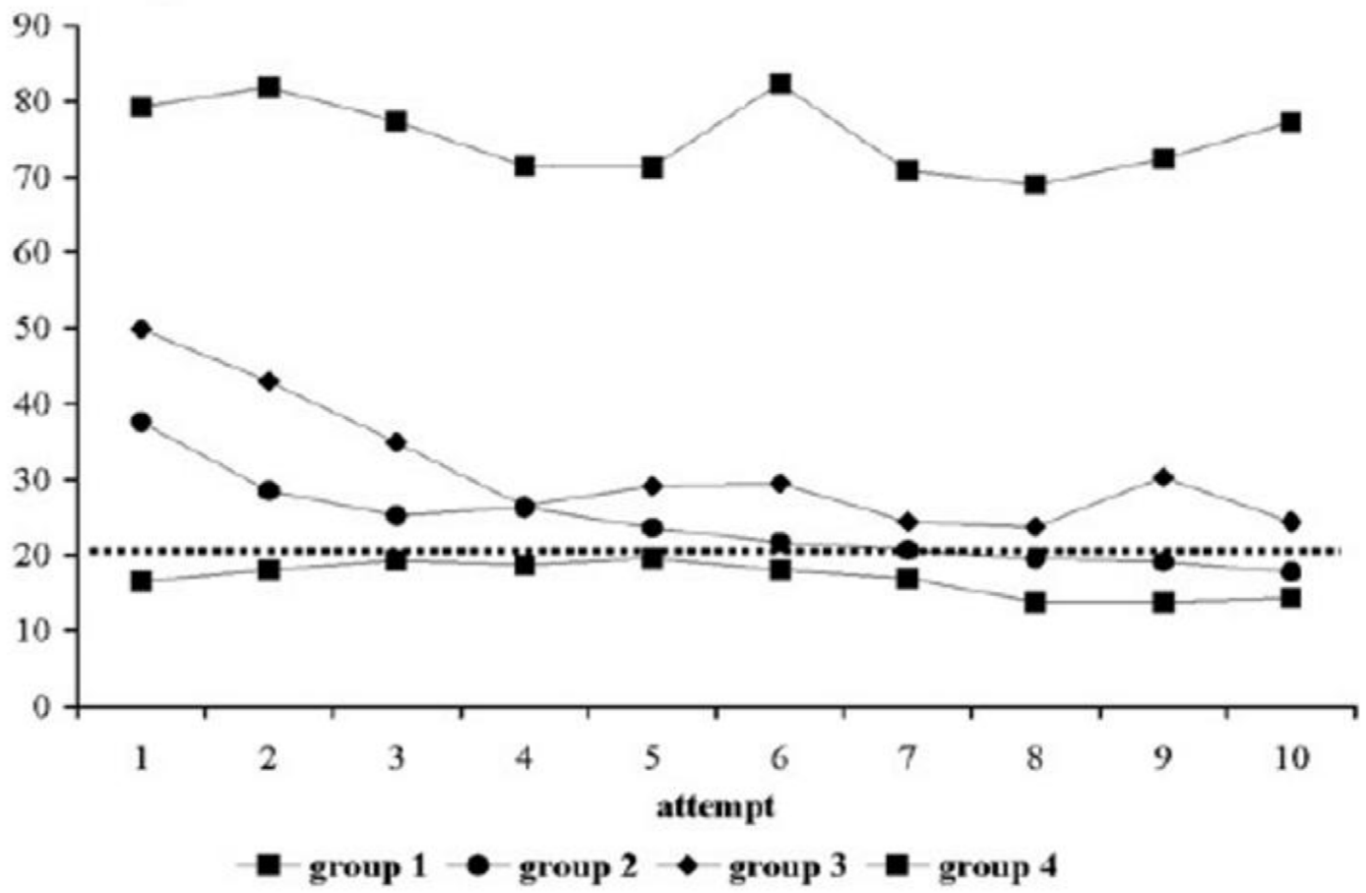 **Figure 7**: *Reproduced with permission from Grantcharov *et al.*, 2009* ( Grantcharov *et al.*, 2009). All groups are surgical residents at different levels performing a task on a laparoscopic simulator stacked in the same graph. The Y-axis is the time to complete the surgical task with each of 10 repetitions with feedback. Note the top group of residents show no time improvement (both qualitatively and quantitatively).
Non-adherence to a typical learning curve shape can suggest that something wrong with the learning context or system.	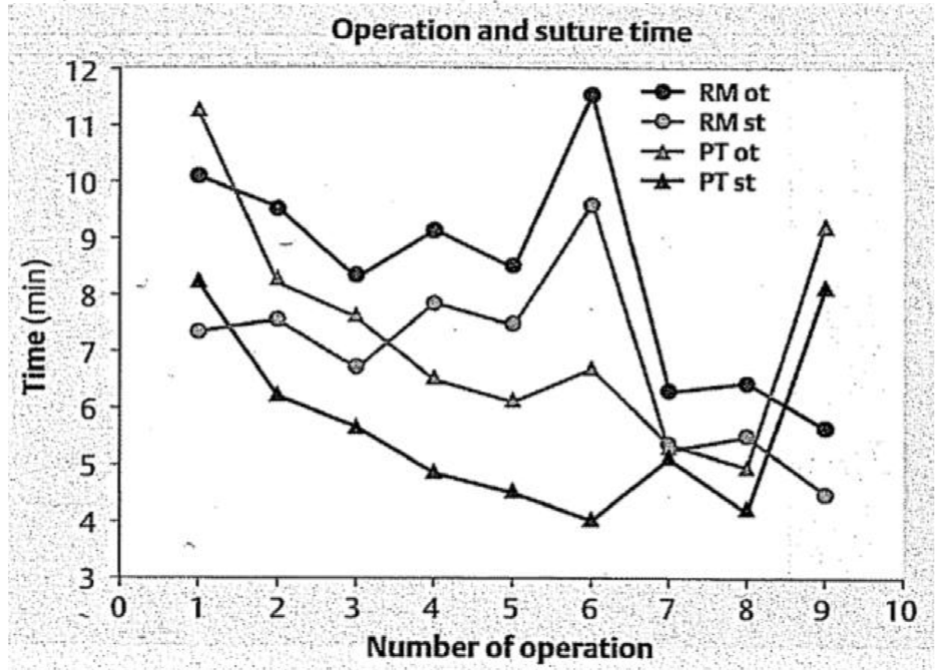 **Figure 8**: *Reproduced with permission from Kirlum, Heinrich, Tillo, & Till, 2005* ( Kirlum *et al.*, 2005). Note that the two triangle groups, although they decreased their operating time on the pelvitrainer (PT) for cases 1-8, this skill was not transferred to the operating room on a rabbit model in operation 9, in comparison to the circle groups who practiced on a rabbit model (RM). This suggests that the learning system (the pelvitrainer) needs to be modified.
Stacked learning curves of different graphs	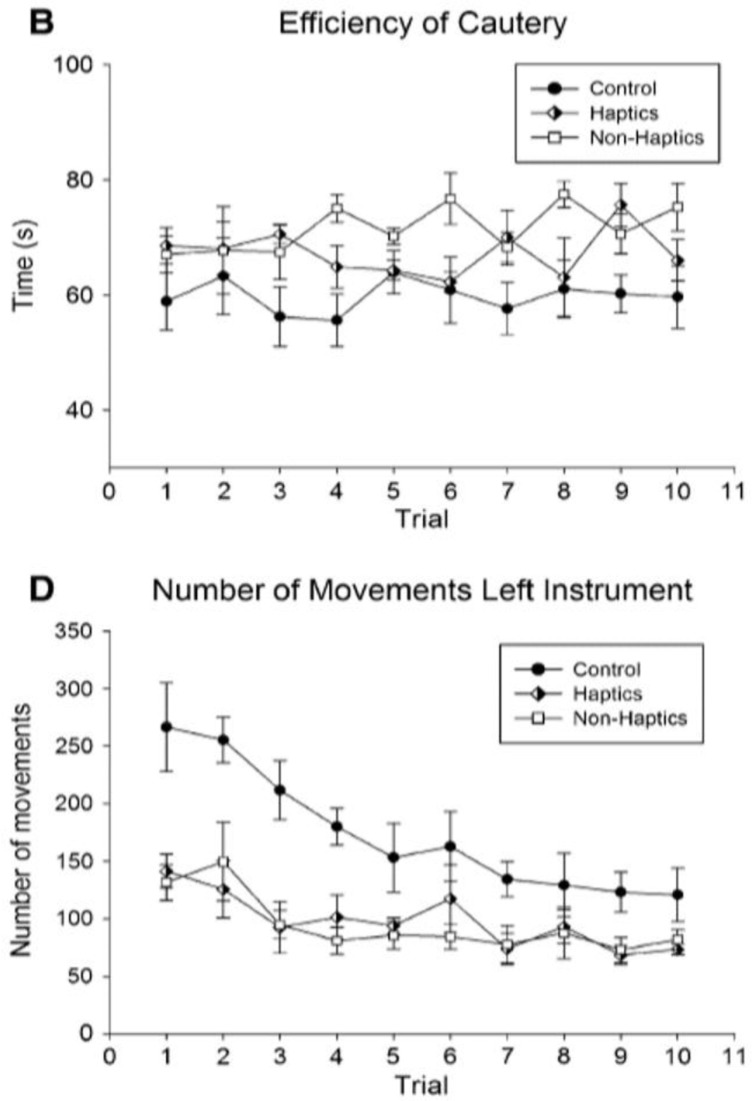 **Figure 9**: *Reproduced with permission from Thompson *et al.*, 2011* ( Thompson *et al.*, 2011). One can grossly intuit that the learning curves in **B** are seemingly random over attempts with a slight increase across the curves, while **D** shows more classically shaped learning curves. These comparisons can provide unique insights into learning outcomes.

## Making decisions with learning curves -- use of thresholds

### Tip 8: articulate a competency threshold and the upper asymptote

When implementing competency-based frameworks, learning curves can inform competency standards since learning curves estimate the amount of learning effort required to achieve a set standard ( Howard *et al.*, 2021). The learning curve’s upper asymptote is helpful in that it represents the theoretical maximum performance boundary for the system or the highest level of expertise within the limits of the learning system ( Pusic *et al.*, 2016). The competency threshold, on the other hand, lies at a value along the Y-axis that defines a consensus minimum level of proficiency. Importantly, this level may have no connection to the inflection point (where learning becomes progressively more difficult) or the asymptote (the theoretical maximum proficiency attainable within the learning system). Determining if a learner has achieved competency in a certain skill, or any measurable property of learning, comes by comparing the learning curve and its associated competency standard ( Figure 1). It will take individuals different numbers of repetitions (X-axis) to reach the competency threshold, but the threshold should be a quantitative measure that students need to achieve to move forward. Multilevel modelling can predict, as mentioned previously (Tips five & six), what amount of time it might take the average learner (or group) to reach this threshold. For example, in Kwan’s *et al.* study of learning curves for POCUS training, individuals achieved an acceptable competency level in vastly different numbers of images practiced ( Kwan *et al.*, 2019). The study underscores that to achieve competency, time must be allowed to vary between learners.

There is ongoing debate regarding the implementation of competencies in HPEd and whether their implementation encourages learners to go beyond the competency threshold to attain expertise ( Elliot & Dweck, 2012; Pugh, 2017). Students tend to rise to the occasion of preset thresholds and not beyond ( Tekian *et al.*, 2015). It is important to consider this juxtaposition when designing curricula; the expectation to regard the competency threshold as a stepping-stone to expertise, rather than the end goal, should be clear. As competencies are defined, definitions for expertise should be presented as well. Comparing student performance to an expertise threshold allows for further room for growth, feedback, and development beyond the level of competency ( Holmboe *et al.*, 2020).

### Tip 9: designate the remediation threshold to show when someone falls off the curve

Another advantage of learning curves is that one can detect when an individual is not adhering to a standard curve and intervene in support of the learner. There will always be multiple paths on the way to competency attainment (See Figure 5, third graph) but early support, or scaffolding from a teacher, can be implemented to optimize a student’s slope of learning. Here again, as in Tip four, the *rate* of learning may be more informative than the absolute value. Where possible plot individual learning curves (Tip six) to understand their variability from the group learning curve. Growth mixed modeling, which identifies trajectories of different subgroups of learners, can be applied to help predict the trajectory of an individual by comparing that individual’s early progress to different empirically derived subgroups ( Mema *et al.*, 2020). You can then predict when remediation is necessary for a student at any point in the system based on their performance relative to different subgroups. This provides an evidence-based means for implementing learning interventions to improve a learner’s overall learning trajectory before they reach their summative end goal.

### Tip 10: address how other variables might affect the shape of the learning curve

Many things can affect learning, and therefore the form of learning curve. These factors fall into the following three categories: intrinsic factors, confounding variables, and distortions. Intrinsic factors are those which are specific to the learning task such as whether a task is easy or difficult to learn. Confounding variables are those that affect the X- or Y-axis variables separately from the linked relationship between the two. For example, if a group of students are bored or disengaged, a learning curve will be artificially flattened ( Pusic *et al.*, 2015). Learning that occurs outside the system can have the opposite effect, making the slope appear more acute. 

Distortions occur when the Y-axis measure is not able to capture the full learning spectrum. For example, a *floor* effect occurs when the Y-variable is not sufficient to be able to demonstrate small increments of learning at the novice end of the performance scale. This can make it appear that the latent phase for learning is prolonged. At the other end, a *ceiling* effect is seen when the Y-variable does not discriminate at the expert end of the scale, making it appear that full expertise has been achieved when in fact we are not able to appreciate ongoing improvements. 

## Putting the learning curve in context

### Tip 11: pay attention to ethics and policy considerations when implementing learning curves

Learning curves are a powerful quantitative tool that provide assessments for learning ( Hatala *et al.*, 2019; Pusic *et al.*, 2015). Learning and its assessment are reflected ultimately in the outcomes of patients, underscoring their importance. They can, however, be misused when designing a curriculum. As a potent tool in formative assessment, use of learning curves should focus on benefitting students who fall off the learning curve so that appropriate interventions can be made. Similar to when an adverse event occurs in a hospital, learning curves provide information on how to improve the educational system, not to blame an individual ( Pusic *et al.*, 2015). Learning curves are inputs to evaluation in a learning and teaching system, not a discriminatory arbiter of individuals’ performances.

Since construction of learning curves involves gathering large amounts of data, ethics regarding data analytics should be considered. To avoid finding patterns in data that are not meaningful, or even harmful, to an individual, all data collection should be guided by a framework that is evidence-based and not discriminatory ( Cirigliano *et al.*, 2017; Lee *et al.*, 2022). For an initial list of ethical questions to consider please see Table 4.

**Table 4.** ethical considerations for the use of learning curves.

Ethical considerations	Sample questions to consider
Learners	What decisions can be made with a learning curve? When is it considered ethical to dismiss a student who does not meet a competency threshold on the learning curve? Can the learning curve be used for formative and customized feedback that is not punitive?
Patients	When a simulator or coach is used to accelerate the learning curve, when can learners start practicing clinical skills directly with patients?
Policy	What guidelines have been implemented prior to incorporating direct patient care into learning curve practice? Have enough resources been allocated to implement effective teaching using learning curves?
Vulnerable populations	If you know a learner is not meeting competency on their learning curve, should they participate in the care of vulnerable populations? Is it possible to provide protection, respect, and dignity to vulnerable populations within an educational environment that still necessitates practice? Are we erring on the side of paternalism with these considerations?

### Tip 12: adhere to best practices for reporting learning curves for dissemination and research

Reporting and use of learning curves in research articles have increased significantly over the last twenty years, however reporting of learning curve elements are often incomplete and their desirable properties discussed here are underutilized ( Howard *et al.*, 2021). Consider including and reporting on as many features as possible, as outlined in Table 5. Expanding your learning curve repertoire will not only offer insights into teaching practices, but also ensure cutting-edge research practices. As an example, we applied all tips to a case study published by Stefanidis *et al*., 2007, “ *Limited feedback and video tutorials optimize learning and resource utilization during laparoscopic simulator training*” (See Table 6).

**Table 5.** Proposed best practices for reporting and use of learning curves in health professions education and research checklist ^*^ *Adapted with permission from Howard *et al.*, 2021* ( Howard *et al.*, 2021).

Basic learning curve feature	Considerations for improved analysis and reporting
Y-axis	○ Show Y-intercept to demonstrate the learners’ starting point(s) and their variability. ○ Show full Y-scale including lowest relevant performance level.
Full path of the learning curve	○ Discuss the shape, which communicates whether the learning is linear or nonlinear. ○ Discuss the efficiency of learning, including comments on the steepness.
Mathematical linking function	○ Include qualitative descriptions. ○ Use parametric linking functions. ○ Take into account the multi-level nature of the data.
X-axis	○ Provide a rationale for the number of data points that are feasible for the task, with spacing clearly reported.
Multiple dimensions of the learning task	○ Show panels of individual learning curves (small multiples, see Figure 4) ○ “Stack” learning curves so that different dimensions can be compared longitudinally, see Table 3
Important thresholds	○ Identify competency or remediation thresholds. ○ Define acceptable individual variations and acknowledge that competence can be redefined as more information is gathered with the learning curve in your setting. ○ Discuss/define the meaning of such thresholds in your educational setting and rationale for use. ○ Use such thresholds as indicators of opportunities to adapt learning when thresholds are not being met.
Asymptote where applicable	○ Include the asymptote, which indicates the maximal learning potential of the system.
Variance in the data	○ Graph data points with corresponding variance. ○ Plot at least a subset of individual learning curves in addition to group curves to demonstrate the variability in paths taken (see Figure 4 & Figure 5).

* Not all of these proposed best practices will be equally relevant in all situations, and in some cases a choice must be made among the options presented.

**Table 6.** As an example, we applied all tips to a case study published by Stefanidis *et al.,* 2007, “ *Limited feedback and video tutorials optimize learning and resource utilization during laparoscopic simulator training*”. What follows are applications of all practical tips using a laparoscopic suturing simulator study.

**Practical tip**	Considerations using a surgical suture simulator example as described in Stefanidis *et al.* (2007). Please read reference and consider Figure 3 in this article for maximum understanding.
**Tip 1: decide if a learning curve can help you in your learning/teaching mission.**	Surgical simulators can be ideal environments in which to use a learning curve as there are many forms of feedback possible (human and machine). They are designed to offer controlled repetitions over time, and data points are straightforward to collect and graph. The deliberate practice environment of simulators offers many opportunities to study learning. If your mission is to offer students a controlled way to learn laparoscopic suture tying, the learning curve can give you information about how many repetitions on the simulator are necessary to reach a defined level of proficiency. If you are trying to answer a research question similar to Stefanidis *et al.,* then the learning curve can shed insight into group comparisons that reflect differing interventions’ effects on learning.
**Tip 2: choose how to represent effort on the X-axis.**	Simulators offer an easily defined X-axis representation of number of repetitions over time on the simulator. Careful consideration of how often a learner completes the repetitions should be considered. Are the learners completing all repetitions in one sitting? Are there practice repetitions between data points? How much time elapses between each data point? All of these factors can have distinct effects on the learning curve that should be explained.
**Tip 3: choose how to represent performance/achievement on the Y-axis.**	In the Stefanidis *et al.* example, the Y-axis performance was an “Objective Score” as defined by a previous validity experiment using the simulator. The advantages of such performance measures are that they take into account many factors, such as time, quality of knots, or tightness of knots. Other measures of performance are often used for knot tying that can demonstrate learning curves such as time per knot, economy of movements (decrease of length of movements over time, or decrease in number of movements over time), failure rate, or collisions with simulator walls (virtual walls of the body cavity). The choice should represent what it is that you want to measure. Always ask yourself, “does my chosen assessment/achievement actually measure what I think it is measuring?” A clue that you are not measuring something that represents learning could be a flat learning curve.
**Tip 4: align your Y-axis with your teaching context or research design.**	Stefanidis *et al.* were interested in comparing different levels of feedback given. Their use of a single Y-axis parameter that encompasses multiple variables in a single score makes sense as they were looking for overall differences between the three feedback conditions. However, you could decide to breakdown each parameter into different learning curves (such as time or economy of movements), but make sure you are looking for a certain hypothesis or interpretation. Economy of movement learning curve differences in addition to Objective Score learning curve differences would likely not add to the feedback story (unless the feedback was specifically related to the variable chosen).
**Tip 5: connect effort with performance.**	Stefanidis *et al.* did not use hierarchical modeling to analyze their data. As a result, they may have lost potentially useful information. They used a fixed effects only non-parametric model, described as ANOVA of ranks – which could have been a Friedman’s or Wilcoxon Signed Ranks method. Their chosen model allowed them to mathematically compare the general shape of the group curves, but not individual-level slopes of the learning curves. They saw no statistical difference between Groups I and II.
**Tip 6: use graphical representations to convey additional learning curve meaning.**	In the Stefanidis *et al.* example (see Figure 3) three learning curves were overlaid on the same graph which allowed immediate visual comparisons between the three groups. Even though statistical analysis was not done comparing the slopes of the curves, visual interpretation makes differences immediately apparent. Stefanidis also stopped collecting data once the proficiency level was met. This allowed for the visual effect of the different stopping points at differing numbers of repetitions to be apparent. One can see that group three achieved proficiency with fewer repetitions.
**Tip 7: find a statistician familiar with hierarchical modeling.**	We cannot speculate on the availability of statisticians with expertise in HLM in 2007 for this group of authors. We would point the reader to tips five and ten in this table for ideas on how HLM could have benefitted this study.
**Tip 8: articulate what the competency threshold and the asymptote represent.**	In this example, proficiency was well articulated as a pre-defined “expert objective score” and was not only drawn on the graph but defined a stopping condition for the experiment. It was not articulated, however, whether further attainment of expertise could push the objective score higher over time. This could be useful to further explain what the objective score actually means in terms of full expertise.
**Tip 9: consider and select the remediation threshold when someone falls off the curve.**	Although the remediation threshold is not indicated in the Stefanidis *et al.* design, consider what you would do if a group never reached proficiency. Would you penalize the students? Or offer a different intervention to “scaffold” or adapt learning to boost the curve to desired levels? These are important considerations to address.
**Tip 10: address hidden variables for a deeper understanding of what is affecting your learning curve.**	When looking at Figure 3, the curves for Groups I and II overlap. Group II has far less total feedback (as measured on a Likert scale) compared to either group I or group III. Is this because Group II was less engaged or bored in the beginning? Boredom or levels of engagement are hidden variables that could be affecting the learning curves. The only way to know is by evaluating these variables and using a hierarchical model to sort out the varying effects.
**Tip 11: pay attention to ethics and policy considerations when implementing learning curves.**	The Stefanidis *et al*. example defines a stopping point for learners in attaining proficiency on the simulator. What does this stopping point mean? Will learners pass an exam or class with this threshold? Does it mean they can implement supervised hands-on patient care? When can learners be unsupervised?
**Tip 12: pay attention to best practices for reporting learning curves for dissemination and research.**	When you are designing your learning intervention or experiment for publication, refer to Table 5 for a checklist of best practices for reporting learning curves. Stefanidis *et al.* met all recommendations except: as discussed prior, they did not use a parametric linking function or consider the multilevel nature of the data (intra-group comparisons such as slopes of the curves were lost). Some possibly valuable information was lost. They indicated that practice and defined feedback for each group occurred in one-hour sessions with the same instructor. However, there was no indication of how many practice sessions were necessary or if there was time between the sessions. How were the sessions spaced? Was it at the convenience of the students or the instructor? The spacing can have large effects on the learning curve and should be included. The asymptote and its meaning were not described, as previously discussed. Variance in the data was not graphed, which could have given further information about whether or not there were enough learners for proper statistical evaluation. Also, individual paths of learners were not graphed at any point, which would have also given us more information about the variance in the data.

## Conclusion

Researchers and educators can improve educational interventions by evaluating the representative learning curve via a multi-faceted framework. Review of definitions from Table 1, visual examples throughout the Tips, and familiarizing oneself with multi-level modeling opens up ideas and applications to enhance the learning experience. Tips 8–12, which discuss making decisions with learning curves, can help quantify the meaning of deliberate practice, competence, and expertise. Using the checklist found in Table 5 ensures readiness to report on the learning curve effectively. Tips 11 & 12 help put your learning curve in the larger context of institutional policy and ethics.

Conceptually, use of learning curves can be applied to enhance all deliberate practice learning across a myriad of educational contexts at the undergraduate, graduate, and even institutional levels ( Pusic *et al.*, 2020). Like a musician or athlete, learners in the health professions can practice using well-honed educational interventions designed to optimize everyone’s personal curve. Fundamentally, learning curves make manifest the goal of rigorous growth and defined pathways to expertise. The enduring message of growth provides the concept of practice to learners, teachers, and institutions on the eternal quest to improve. The rigorous implementation of the learning curve provides the necessary framework.

## Data Availability

No data are associated with this article.
