# Supplementary material for: Twelve Tips for using Learning Curves in Health Professions Education Research
Source: MedEdPublish (2016). 2023 Nov 7;13:269. [Version 1] doi: 10.12688/mep.19723.1 (PMC10696298; doi:10.12688/mep.19723.1)
Supplement: Peer review history

# Reviewer report: Reviewer response for version 1

**DOI:** 10.21956/mep.21130.r35421

**Published:** 2023-12-04

**Authors:** Kenneth Masters

**Affiliations:** 1 Medical Education and Informatics Department, College of Medicine and Health Sciences, Sultan Qaboos University, Muscat, Oman

This is a really detailed paper on the application of Learning Curves to HPE. The opening section gives a good theoretical background, and the tips follow the process in a logically-ordered manner, giving examples of application. Because the details could become a little overwhelming, I think most readers will appreciate the initial Table 1 giving the definitions and their placement in the paper, and then the worked example of applying the concept at the end of the paper.  The authors are also careful to show the value of learning curves, without over-stating their case, and they apply reasonable warnings, especially on appropriate use and contextualisation.

 I have no major criticisms of the paper. 

 I do have two minor points, both dealing with wording in the introduction. It’s probably not essential to address these, but it may be worth tidying up to not have these small issues detract from the value of the paper.

- The authors may wish to adjust their commentary on the general use of the term “learning curve”. The authors write: “First consider a “steep learning curve”, which is colloquially meant to refer to a skill which is difficult to master in the way a steep hill would be difficult to climb.” That interpretation does exist, as is showing on https://www.valamis.com/hub/steep-learning-curve where that is expressed, and the same metaphor is used. However, that is only one interpretation, and not necessarily the most common, as indicated at https://samelane.com/blog/what-is-a-steep-learning-curve/  “A steep learning curve refers to a situation where a person is expected to learn a new skill or knowledge rapidly, often in a short period.”  This is more in line with the learning curve as described by the authors.
- Similarly, “colloquially referred to as the law of diminishing returns”. Do the authors mean when referring to this model only, or in general? I ask that because the “law of diminishing returns” is not really a colloquial expression, as implied in the paper; it is a fairly-well established principle applied in economics and elsewhere. 

An interesting read, and I would like to see other researchers follow up with applications to their work, especially longer-term studies or full courses.

If evidence from practice is presented, are all the underlying source data available to ensure full reproducibility?

Not applicable

Is the topic of the practical tips discussed accurately in the context of the current literature

Yes

Are all factual statements correct and adequately supported by citations?

Yes

Are the conclusions drawn balanced and justified on the basis of the presented arguments?

Yes

Are arguments sufficiently supported by evidence from the published literature and/or the authors’ practice?

Yes

Reviewer Expertise:

Medical education

I confirm that I have read this submission and believe that I have an appropriate level of expertise to confirm that it is of an acceptable scientific standard.

# Reviewer report: Reviewer response for version 1

**DOI:** 10.21956/mep.21130.r35260

**Published:** 2023-12-04

**Authors:** Raquel Correia

**Affiliations:** 1 Université de Paris Cité, Paris, France

This paper is an insightful contribution to the field of HPE. It elucidates the use of learning curves in educational research and practice. The authors offer practical tips on effectively utilizing these curves, emphasizing their role in designing, implementing, and evaluating educational interventions. They also discuss the ethical and policy considerations associated with learning curves, culminating in a checklist of best practices for educators and researchers. I really appreciate that the authors have made an effort to make their methodology and data clear, facilitating further research in this area.

 On this note, a future paper discussing potential areas for future research based on the current findings would be valuable as this is an expanding field in HPE.

If evidence from practice is presented, are all the underlying source data available to ensure full reproducibility?

Yes

Is the topic of the practical tips discussed accurately in the context of the current literature

Yes

Are all factual statements correct and adequately supported by citations?

Yes

Are the conclusions drawn balanced and justified on the basis of the presented arguments?

Yes

Are arguments sufficiently supported by evidence from the published literature and/or the authors’ practice?

Yes

Reviewer Expertise:

VBHC, TEL, CPD

I confirm that I have read this submission and believe that I have an appropriate level of expertise to confirm that it is of an acceptable scientific standard.

# Reviewer report: Reviewer response for version 1

**DOI:** 10.21956/mep.21130.r35258

**Published:** 2023-11-14

**Authors:** John Sandars

**Affiliations:** 1 Edge Hill University, England, UK

This article provides an excellent overview of the use of learning curves in HPE. The practical advice is supported by the extensive experience of the authors and also by appropriate theory and evidence. Important aspects of the usefulness of the article are the clear explanation of key concepts, numerous informative figures and tables, and discussion of the practical challenges of using learning curves in HPE, including the essential ethical aspects.

If evidence from practice is presented, are all the underlying source data available to ensure full reproducibility?

Not applicable

Is the topic of the practical tips discussed accurately in the context of the current literature

Yes

Are all factual statements correct and adequately supported by citations?

Yes

Are the conclusions drawn balanced and justified on the basis of the presented arguments?

Yes

Are arguments sufficiently supported by evidence from the published literature and/or the authors’ practice?

Yes

Reviewer Expertise:

Use of technology and professional performance assessment, including the use of self-regulated learning and personalised learning.

I confirm that I have read this submission and believe that I have an appropriate level of expertise to confirm that it is of an acceptable scientific standard.
